# Functional coupling between large-conductance potassium channels and Cav3.2 voltage-dependent calcium channels participates in prostate cancer cell growth

**DOI:** 10.1242/bio.20135215

**Published:** 2013-07-26

**Authors:** Florian Gackière, Marine Warnier, Maria Katsogiannou, Sandra Derouiche, Philippe Delcourt, Etienne Dewailly, Christian Slomianny, Sandrine Humez, Natalia Prevarskaya, Morad Roudbaraki, Pascal Mariot

**Affiliations:** Laboratoire de Physiologie Cellulaire, INSERM U1003, Bâtiment SN3, Université Lille 1, 59655 Villeneuve d'Ascq Cédex, France

**Keywords:** BK channels, KCa1.1, Cav3.2, CACNA1H, T-type calcium channels, Proliferation, Prostate, Cancer cell growth

## Abstract

It is strongly suspected that potassium (K^+^) channels are involved in various aspects of prostate cancer development, such as cell growth. However, the molecular nature of those K^+^ channels implicated in prostate cancer cell proliferation and the mechanisms through which they control proliferation are still unknown. This study uses pharmacological, biophysical and molecular approaches to show that the main voltage-dependent K^+^ current in prostate cancer LNCaP cells is carried by large-conductance BK channels. Indeed, most of the voltage-dependent current was inhibited by inhibitors of BK channels (paxillin and iberiotoxin) and by siRNA targeting BK channels. In addition, we reveal that BK channels constitute the main K^+^ channel family involved in setting the resting membrane potential in LNCaP cells at around −40 mV. This consequently promotes a constitutive calcium entry through T-type Cav3.2 calcium channels. We demonstrate, using single-channel recording, confocal imaging and co-immunoprecipitation approaches, that both channels form macromolecular complexes. Finally, using flow cytometry cell cycle measurements, cell survival assays and Ki67 immunofluorescent staining, we show that both BK and Cav3.2 channels participate in the proliferation of prostate cancer cells.

## Introduction

Ion channels have been shown to be implicated in several aspects of cancer development in various organs including the prostate (Fiske et al., 2006; Kunzelmann, 2005). These include sodium channels in cell invasion and migration (Bennett et al., 2004), voltage-dependent calcium (Ca^2+^) channels in neuroendocrine differentiation (Mariot et al., 2002), non voltage-dependent TRP and ORAI Ca^2+^ channels in migration or proliferation (for a review, see Prevarskaya et al., 2011) or potassium (K^+^) channels in cell proliferation (Skryma et al., 1999; Spitzner et al., 2007). The largest family of membrane ion channels, namely K^+^ channels, has been shown to be involved in cell proliferation. Some of these are Ca^2+^-dependent K^+^ channels (Lallet-Daher et al., 2009), others are KATP channels (Huang et al., 2009), KNCQ channels (Morokuma et al., 2008), or EAG channels (Pardo et al., 1999). Indeed, EAG channels have been proposed as tumoral markers (Farias et al., 2004) and clinical targets (Pardo and Sühmer, 2008). In androgen-sensitive prostate cancer LNCaP cells, it has been shown that blocking K^+^ channel activity with inhibitors such as tetraethyl ammonium (TEA) reduced cell growth (Skryma et al., 1997). However, the mechanisms through which K^+^ channels regulate cell growth have scarcely been described. Several hypotheses have been put forward, involving either decreases in cytosolic K^+^ concentration, membrane hyperpolarization, cytosolic Ca^2+^ increases, or pH variations (Lang et al., 2005; Spitzner et al., 2007). Regarding prostate cancer LNCaP cells, recent studies conducted in our laboratory have demonstrated that intermediate IK potassium channels (also called IKCa1, IK1 or KCa3.1) are functional and regulate cell proliferation (Lallet-Daher et al., 2009). In addition, it has been shown by others that Ca^2+^- and voltage-dependent BK potassium channels (also called BK_Ca_ or KCa1.1) are responsible for large K^+^ currents in LNCaP cells (Yan and Aldrich, 2010).

In the present study, we wished to assess the mechanisms through which voltage-dependent K^+^ channels could regulate LNCaP cell growth. Since voltage-dependent Ca^2+^ channels have also been shown to be expressed in LNCaP cells, we hypothesized that both voltage-dependent K^+^ and Ca^2+^ channels in close association, could co-regulate cell proliferation. Our experiments using pharmacological and molecular evidences, confirm previous works (Yan and Aldrich, 2010), namely that most of the voltage-dependent K^+^ current in LNCaP cells is carried by BK channels. We show that BK channels in LNCaP cells may open at resting membrane potential, even in conditions of low cytosolic Ca^2+^ concentrations. However, they are still sensitive to Ca^2+^, since their open probability may be increased by raising cytosolic Ca^2+^ either with an intracellular perfusion of high concentrations of Ca^2+^, or by activating Ca^2+^ entry. We show that membrane depolarizations increased BK channel activity in LNCaP cells expressing Cav3.2 T-type Ca^2+^ channels. Furthermore, a transient Ca^2+^ entry through Cav3.2 channels is able to induce a persistent BK channel activation. We demonstrate using single-channel recording, confocal imaging and co-immunoprecipitation approaches, that T-type channels and BK channels are located in the same patches of membranes, probably forming functional complexes. Finally, using specific inhibitors and siRNA, we show that BK and Cav3.2 channels set the resting membrane potential in prostate cancer cells and thereby participate in cell proliferation. These results highlight that there is a functional coupling in LNCaP cells between BK channels and Cav3.2 channels and that this coupling may participate in prostate cancer cell growth.

## Results

**Fig. 1.**
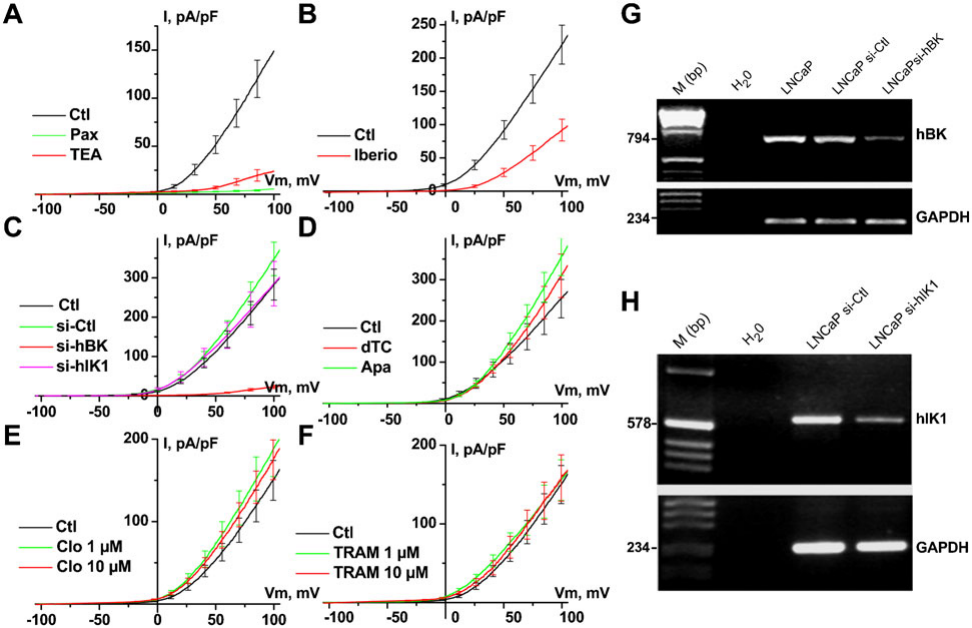
Blocking BK channels inhibits voltage-dependent K^+^current in LNCaP-CTL cells. (**A–F**) Current–voltage (i–v) relationships in the presence of different K^+^ channel inhibitors. Concentrations used were: 4 mM TEA, 1 µM paxillin (Pax), 1 µM iberiotoxin (Iberio), 500 nM apamin (Apa), 100 µM d-tubocurarine (dTC), 1 or 10 µM clotrimazole (Clo), 1 or 10 µM TRAM-34 (TRAM). Treatments with different siRNAs (si-hBK, si-Ctl, si-hIK1, 20 nM) were carried out for 3–4 days. (**G**) RT-PCR showing a decrease in the expression of the BK channel amplicon following 3 days of treatment with si-hBK (20 nM). Lanes correspond to: H_2_O  =  negative control, LNCaP  =  sample from LNCaP-CTL cells, LNCaP si-Ctl  =  sample from LNCaP-CTL cells treated with 20 nM si-Ctl, LNCaP si-hBK  =  sample from LNCaP-CTL treated with 20 nM si-hBK. Expression of hBK was compared to that of GAPDH. (**H**) RT-PCR showing a decrease in the expression of the hIK1 channel amplicon following 3 days of treatment with si-hIK1 (20 nM). Lanes correspond to: H_2_O  =  negative control, LNCaP si-Ctl  =  sample from LNCaP-CTL cells treated with 20 nM si-Ctl, LNCaP si-hIK1  =  sample from LNCaP-CTL treated with 20 nM si-hIK1. Expression of hIK1 was compared to that of GAPDH.

LNCaP cells display large voltage-dependent K^+^ currents, which are activated at around −10 mV when Ca^2+^ is buffered with 1–10 mM EGTA in the patch-pipette (Fig. 1). As shown in Fig. 1, TEA (10 mM) almost totally inhibited voltage-dependent K^+^ currents (*n* = 20). The activity of known BK channel inhibitors (paxillin, iberiotoxin (Ghatta et al., 2006; Sanchez and McManus, 1996)), IK channel inhibitors (TRAM-34, clotrimazole) and SK channel inhibitors (apamin, d-tubocurarine) was thus assessed on these currents. Paxillin totally inhibited voltage-dependent K^+^ currents (Pax, 1 µM, *n* = 19), whereas iberiotoxin only reduced them (Iberio, 1 µM, *n* = 10). In contrast, neither TRAM-34 (TRAM, 1–10 µM, *n* = 25), clotrimazole (Clo, 1–10 µM, *n* = 25), d-tubocurarine (dTC, 100 µM, *n* = 8) nor apamin (Apa, 500 nM, *n* = 8) reduced voltage-dependent K^+^ currents. In addition, whole-cell voltage-dependent K^+^ currents were strongly inhibited by siRNA targeted against BK channels (si-hBK), but not by siRNA against IK channels (si-hIK1) (Fig. 1C). Si-RNA targeted against BK or IK channels were validated by RT-PCR as shown in Fig. 1 (G and H, respectively).

**Fig. 2.**
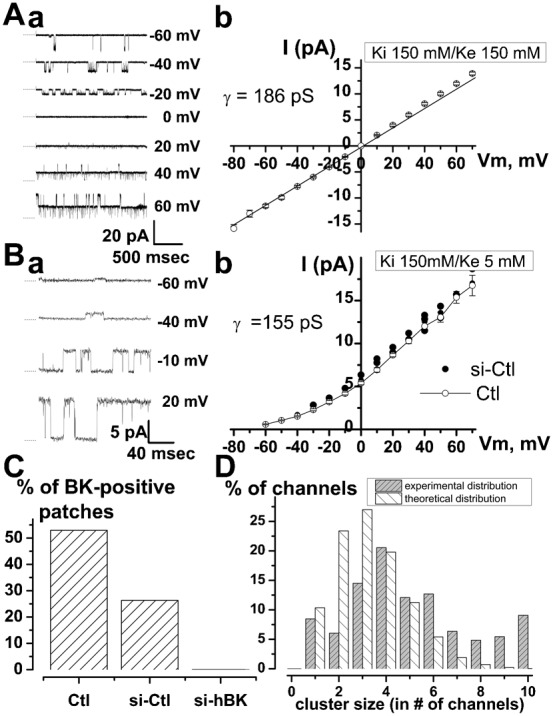
Single-channel characterization of voltage-dependent K^+^ channels in LNCaP-CTL cells. (**A**) (**a**) Outside-out patch-clamp recording of BK channels openings at different membrane potentials in symmetrical K^+^ concentration (150 mM). (A) (**b**) Corresponding i–v curve in symmetrical K^+^ concentration (150 mM). (**B**) (**a**) Outside-out patch-clamp recording of BK channels openings at different membrane potentials in asymmetrical K^+^ concentration (150 mM in the pipette, 5 mM in the bath). (B) (**b**) Corresponding i–v curve in asymmetrical K^+^ concentration. (**C**) Proportion of outside-out patches displaying BK channel activity in the absence of siRNAs, after 3 days of treatment in the presence of ctl siRNA (si-Ctl, 20 nM) or in the presence of si-hBK (20 nM). (**D**) Proportion of BK channels (*n* = 331) expressed in the plasma membrane is shown as a function of cluster size (for example, 20% of all BK channels were observed in patches containing 4 levels of opening – i.e in cluster size 4). 31% of the patches were devoid of any channel activity. Theoretical stochastic binomial distribution is also shown. Formula used to compute binomial distribution is:
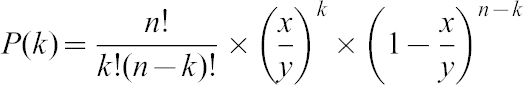
where *P*(*k*) is the probability for one channel of belonging to a cluster of *k* channels in a membrane patch of 2 µm^2^ surface, *n*  =  total number of BK channels on the plasma membrane (estimation  =  6500), *x*  =  patch area (estimated to 2 µm^2^ from the patch-pipette size), *y*  =  plasma membrane area (estimation  =  6500 µm^2^). Here, the binomial distribution represents *P*(*k*)*k as a function of *k* (in percentage).

**Fig. 3.**
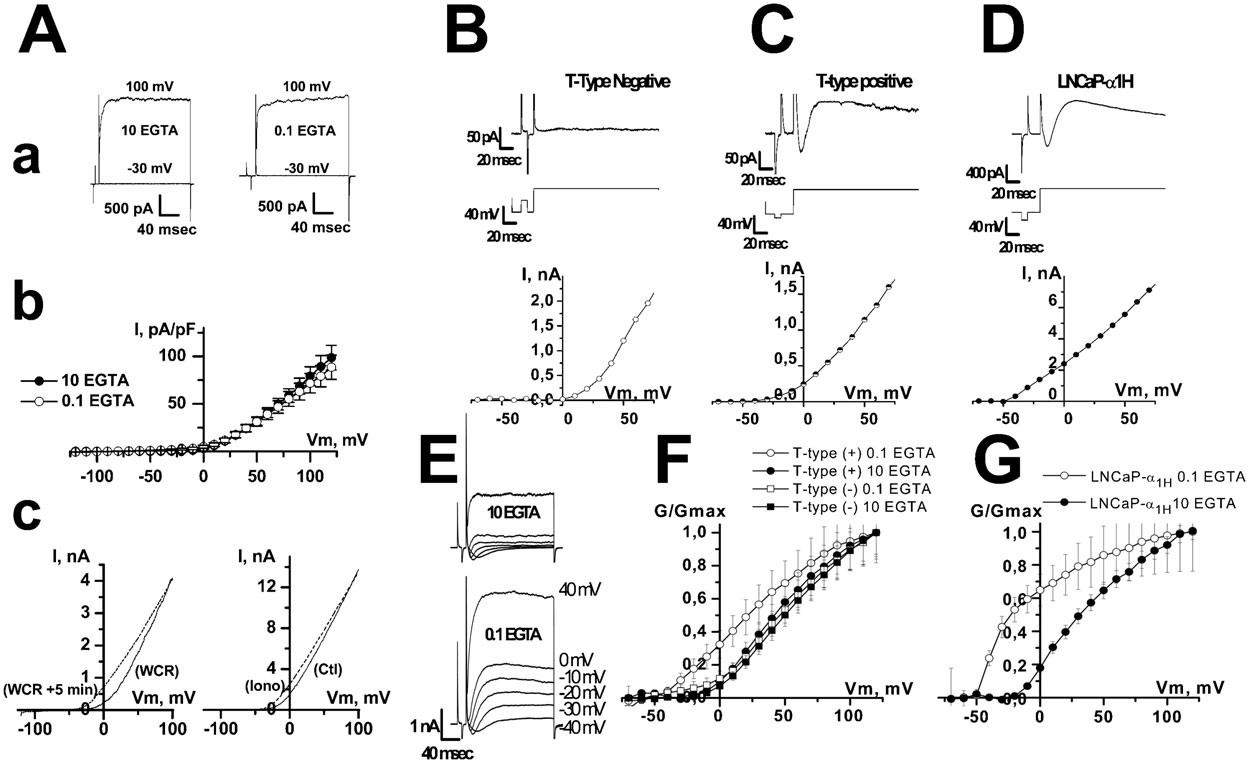
Comparison of voltage-dependent K^+^ current in LNCaP cells with T-type Ca^2+^ current different expression levels (A) Ca^2+^-dependency of voltage-dependent K^+^ current in LNCaP-CTL cells. (**A**) (**a**) Typical membrane currents at −30 and +100 mV in the presence of either 10 or 0.1 mM EGTA in the patch-pipette. (A) (**b**) Average i–v curves obtained in the presence of either 10 or 0.1 mM EGTA in the patch-pipette. (A) (**c**) Typical i–v curves obtained using ramp protocols show that increasing intracellular Ca^2+^ concentration shifts the i–v curve towards negative potentials. Left panel: intracellular perfusion of 400 nM Ca^2+^. Perfusing high concentration of Ca^2+^ into the cells was carried out using an EGTA-buffered solution in the patch-pipette (10 mM EGTA, 6.5 mM CaCl_2_ and 1 mM MgCl_2_). After breaking into whole-cell configuration, this solution shifted the i–v curve towards negative membrane potentials (WCR: current recorded just after breaking into whole-cell recording configuration, WCR+5 min: current recorded 5 minutes later). Right panel: bath perfusion with ionomycin (Iono, 1 µM), results in an increased K^+^ current and its shift to more negative membrane potentials. (**B**) In LNCaP-CTL cells that do not display any T-type Ca^2+^ current, no K^+^ current was observed for membrane potentials lower than 0 mV. Top panel: membrane current. Middle panel: pulse protocol. Bottom panel: I/V curve. (**C**) In LNCaP cells that express T-type Ca^2+^ current, here a LNCaP-NE cell, this transient Ca^2+^ current was followed by potassium current that could be observed for membrane potential ranging from −40 to 0 mV. (**D**) Similar results were observed for LNCaP cells stably overexpressing Cav3.2 channels (LNCaP-α_1H_). (**E**) In LNCaP-α_1H_ cells, the K^+^ current was larger when EGTA was reduced in the patch-pipette (0.1 *vs* 10 mM EGTA). (**F**) Representation of relative membrane conductance (G/Gmax) in LNCaP cells displaying (T-type (+)) or not (T-type (−)) T-type Ca^2+^ current with 0.1 and 10 mM EGTA in the patch-pipette. (**G**) Representation of the relative membrane conductance (G/Gmax) in LNCaP-α_1H_ cells with 0.1 and 10 mM EGTA in the patch-pipette.

Single-channel experiments carried out in the outside-out patch-clamp configuration allowed us to measure the single channel activity (Fig. 2) corresponding to this voltage-dependent K^+^ current. In symmetrical K^+^ condition (150 mM K^+^ on each side of the patch), channel activity displayed a linear current-voltage (i–v) relationship with a reversal potential of 0 mV and an average conductance of 186±2.7 pS (Fig. 2A). In asymmetrical K^+^ condition (5 mM K^+^ on the outer side of the patch *vs* 150 mM on the inner side of the patch), the i–v relationship was no longer linear and displayed a slight outward rectification. The average conductance, which was measured in the linear part of the i–v curve (between −10 mV and 60 mV), was 155±3.9 pS in LNCaP cells (*n* = 15) and 146±4.7 pS (*n* = 5) in LNCaP cells treated with si-Ctl (Fig. 2B). The reversal potential shifted to values close to −80 mV, thus confirming that these are indeed K^+^ channels. Such properties, including a large conductance, are characteristic features of BK channels. Whereas a treatment with si-Ctl did not significantly change the percentage of cell patches displaying a BK channel activity (5 out of 19 for si-Ctl cells *vs* 9 out of 17 for Ctl cells, non significant, Fisher's test), si-hBK completely inhibited (21 out of 21), the occurrence of this channel activity (Fig. 2C). The number of BK channels in a patch was estimated from the number of openings observed at a membrane potential for which the maximal open probability was observed (usually +20 mV). BK channel density did not vary (*P*>0.05) between different LNCaP cell lines (2±0.3 BK channels per patch for LNCaP-CTL cells (*n* = 55), 2.6±0.3 for LNCaP-NE cells (*n* = 32), 2.8±0.5 for LNCaP-α_1H_ cells (*n* = 50)). Altogether, we observed that 31% of the patches were devoid of any BK channels and 21% of the patches displayed only one opening level. As shown in Fig. 2D, which represents the proportion of the recorded BK channels in this study (*n* = 137 patches, 311 channels) as a function of the cluster size (number of channels in a patch), most of the BK channels were present in clusters on the plasma membrane. A theoretical stochastic distribution of BK channels (following a binomial distribution) on the plasma membrane should lead to a different distribution as shown in Fig. 2D. As an example, 38.3% of all the BK channels are located in clusters of 6 channels or more, whereas a binomial distribution would give a figure of 8.3% (significantly different, *P*<0.005). With an average figure of 2.3±0.3 BK channels per patch (*n* = 137 patches, 311 channels), we estimated that the density of BK channels on the plasma membrane was around 1 per µm^2^, which means about 6500 channels for an average LNCaP cell (mean capacitance 65±3 pF). As shown on Fig. 3A, although such BK channels may be activated in the absence of Ca^2+^ in the patch-pipette (10 mM EGTA), perfusing high concentrations of Ca^2+^ into the cells (400 nM) shifted the i–v curve towards negative membrane potentials (Fig. 3A). Ionomycin (1 µM), a Ca^2+^ ionophore, similarly induced a potentiation of BK currents (Fig. 3A), further inhibited by paxillin and si-hBK.

Since BK channels are activated by cytosolic Ca^2+^, we investigated whether the activation of Ca^2+^ channels in LNCaP cells could lead to the activation of BK channels. As previously shown, T-type Ca^2+^ currents are expressed in about 30% of LNCaP-CTL cells and 80% of LNCaP-NE cells (Mariot et al., 2002). In LNCaP cells overexpressing Cav3.2 channels (LNCaP-NE cells or LNCaP-α_1H_ cells), paxillin (*n* = 34) and si-hBK (*n* = 54) almost totally inhibited whole-cell BK currents, as was the case in LNCaP-CTL cells. When T-type Ca^2+^ currents were undetectable, depolarization protocols led to outward currents at membrane potentials positive to −10 mV (Fig. 3B). When T-type Ca^2+^ currents were detectable, depolarization protocols led to inward Ca^2+^ currents followed by outward currents at membrane potentials positive to −50 mV (LNCaP-α_1H_ cells, in 100% of the cells, *n* = 363, Fig. 3D) and −40 mV (LNCaP-NE cells, in 84% of the cells, *n* = 224; LNCaP-CTL cells, in 32% of the cells, *n* = 81, Fig. 3C) in the presence of 0.1 mM EGTA in the patch-pipette. These T-type-activated BK currents had low inactivating kinetics (τ = 167±35 msec in LNCaP-NE cells and 5090±900 msec in LNCaP-α_1H_ cells), which were not altered by treatments with ruthenium red (10–100 µM, *n* = 10–19), heparine (5 mg/ml, *n* = 19) and Xestospongin C (5 µM, *n* = 11) in the patch-pipette, or by ryanodin (10–100 µM, *n* = 17) in the external medium. This would show that they probably do not involve a Ca^2+^-induced Ca^2+^ release (not shown). Using tail-current protocols, we noted that these T-type-activated BK currents reversed at −81±2 mV in LNCaP-CTL, LNCaP-NE and LNCaP-α_1H_ cells (not shown). T-type-activated BK currents were inhibited by concentrations of 10 mM EGTA a few minutes after breaking into whole-cell configuration, in both LNCaP-NE (*n* = 12) and LNCaP-α_1H_ cells (*n* = 10) (Fig. 3E–G).

**Fig. 4.**
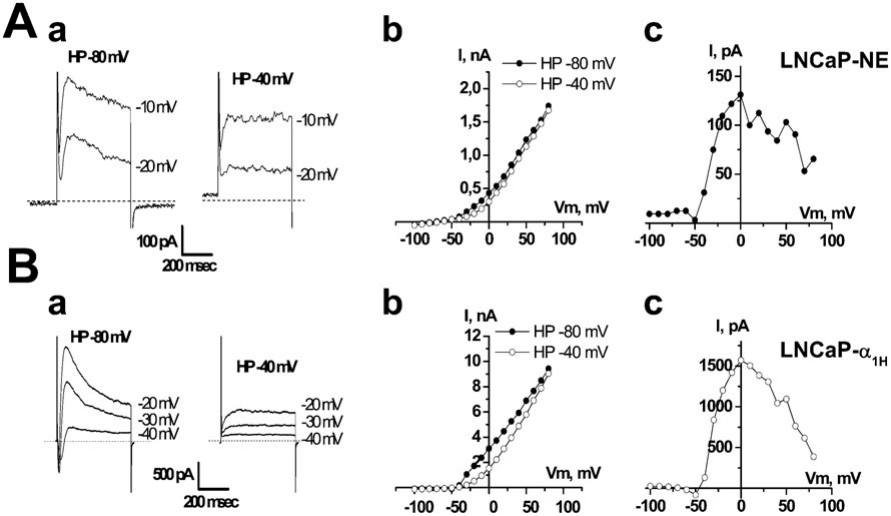
The transient component of voltage-dependent K^+^ current is inhibited by depolarizing the holding potential (HP) from −80 to −40 mV in (A) LNCaP-NE and (B) LNCaP-α_1H_ cells. (**A**,**B**) (**a**) Examples of membrane currents triggered by voltage steps to various membrane potentials from two different HP (−80 and −40 mV). (**b**) i–v curves obtained from HP of −80 and −40 mV. (**c**) i–v curves displaying the difference between the current measured at HP −80 mV and that measured at HP −40 mV.

**Fig. 5.**
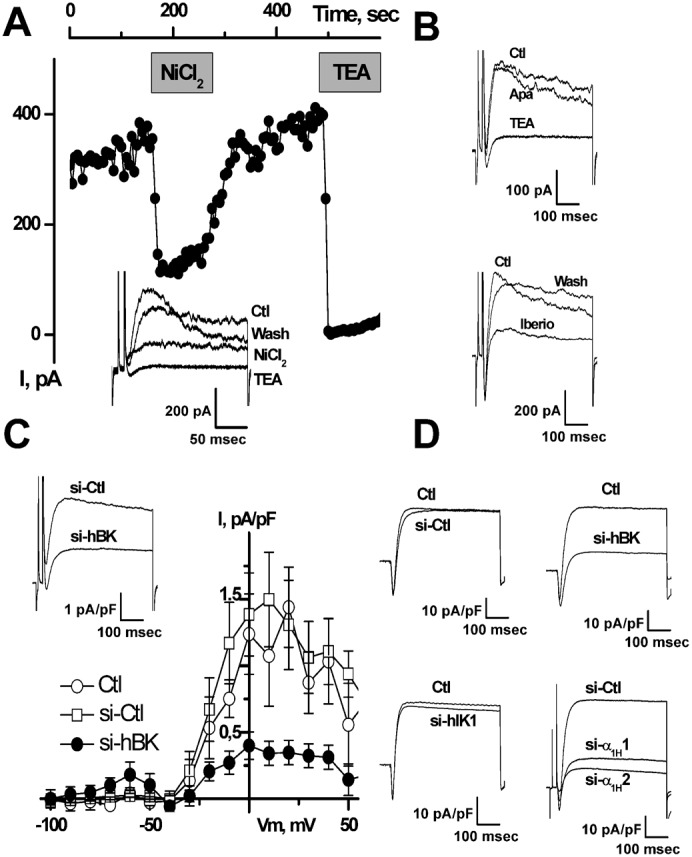
Pharmacological study of the transient voltage-dependent K^+^ current in LNCaP-NE cells (A,B,C) and LNCaP-α_1H_ cells (D). (**A**) On-line recording of transient voltage-dependent K^+^ currents inhibition by NiCl_2_ (10 µM) and TEA-Cl (20 mM). Inset: representative membrane currents measured at −20 mV from HP −80 mV. (**B**) Inhibition of membrane currents (measured at −20 mV from HP −80 mV) by TEA (20 mM) and iberiotoxin (Iberio, 1 µM), but not by apamin (Apa, 500 nM). (**C**) Inhibition of voltage-dependent K^+^ current by si-hBK (20 nM). i–v curves shown here represent the average difference between currents obtained at HP −80 mV and those obtained at HP −40 mV. Inset: representative membrane currents measured at −20 mV from HP −80 mV. (**D**) Representative inhibition of membrane currents (measured at −20 mV from HP −80 mV) by si-hBK (20 nM) and si-α_1H_1 and si-α_1H_2 (5 nM), but not by si-Ctl (20 nM) or si-hIK1 (20 nM). Treatments for 3 days with si-hBK (20 nM) inhibit about 80% of the Ca^2+^-dependent K^+^ current in both LNCaP-NE (**C**) and LNCaP-α_1H_ (**D**) cells.

In addition, a conditioning pre-pulse at −40 mV, which induces full inactivation of T-type Ca^2+^ channels, completely inhibited T-type-activated BK currents (Fig. 4). This shows that Ca^2+^ entry through T-type Ca^2+^ channels can activate BK currents. The substraction of K^+^ currents obtained at a holding potential (HP) of −40 mV from the total K^+^ currents obtained at an HP of −80 mV, gave bell shaped i–v curves, similar to those of T-type Ca^2+^ currents (Fig. 4Ac,Bc). Furthermore, inhibitors of T-type Ca^2+^ channels such as NiCl_2_ (10–100 µM, *n* = 16–14, Fig. 5), mibefradil (5 µM), flunarizine (5 µM) or kurtoxin (100 nM) inhibited both inward T-type currents and T-type-activated BK currents in LNCaP-CTL, LNCaP-α_1H_ and LNCaP-NE cells. However, the purely voltage-dependent component of the BK current was not altered by these inhibitors.

T-type-activated BK currents were 50% inhibited for a concentration of about 1–2 mM TEA (IC_50_ = 1.9±0.7 mM and 1.14±0.2 mM, for LNCaP-NE and LNCaP-α_1H_ cells, respectively). In addition, they were inhibited in both LNCaP-NE and LNCaP-α_1H_ cells (Fig. 5) by paxillin (1 µM) or iberiotoxin (1 µM), but not by apamin (500 nM, *n* = 4), d-tubocurarine (100 µM, *n* = 4), or clotrimazole (10 µM, *n* = 7). As shown in Fig. 5C,D, downregulation of BK channels by si-hBK decreased the magnitude of the T-type activated BK currents in both LNCaP-NE and LNCaP-α_1H_ cells. In addition, knocking down the expression of Cav3.2 Ca^2+^ channels (α_1H_ T-type Ca^2+^ channels) with previously validated siRNAs (si-α_1H_1 and si-α_1H_2 (Gackière et al., 2008)), considerably reduced the amplitude of T-type-activated BK currents (Fig. 5D). Conversely, si-hIK1 and si-Ctl had no significant influence on T-type Ca^2+^ currents or on T-type-activated BK currents.

**Fig. 6.**
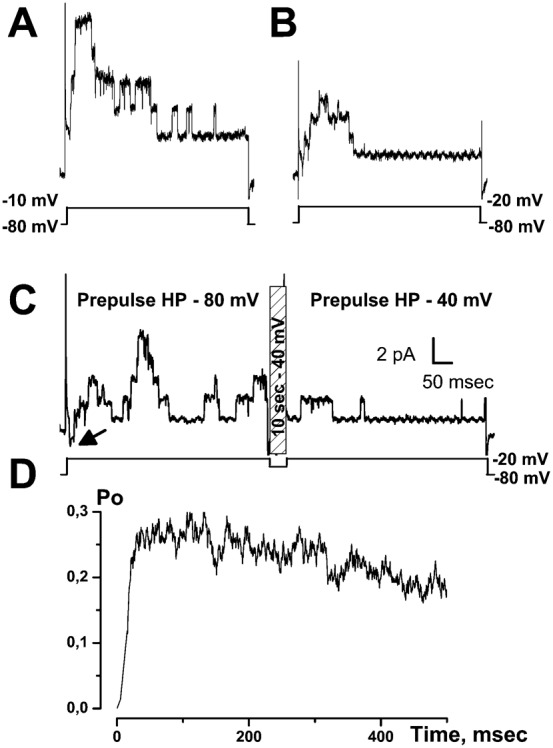
Cell-attached single-channel study of coupling between BK and Cav3.2 channels in LNCaP-NE cells. (**A**) Example of recording of BK channel opening following a voltage step to −10 mV. (**B**) Example of recording of BK channel opening following a voltage step to −20 mV. As seen, channel opening occurs mainly at the beginning of the depolarization. (**C**) Examples of channel openings following stimulation to −20 mV for two different holding potentials (HP). For each patch, the same protocol was applied (shown below channel recordings). The patch was first depolarized from a HP of −80 mV (pre-pulse HP) to a test pulse of −20 mV for 500 msec (left panel). The membrane potential was then immediately returned to a HP of −40 mV for 10 sec in order to inactivate T-type Ca^2+^ channels. A second test pulse to −20 mV was then applied for 500 msec (right panel). The membrane potential was then returned to −80 mV. As seen, BK channel opening for a HP of −80 mV occurs immediately after the inward Ca^2+^ current (arrow). This is impeded when an HP −40 mV pre-pulse was applied for 10 sec just before the test pulse. The scale applies to panels A–C. (**D**) Average BK channel open probability (Po) during a depolarization to −20 mV in LNCaP-NE cells. This value is computed by averaging (20–50 different voltage pulses from 15 cells) and subtracting single-channel currents obtained for an HP of −40 mV from those obtained for an HP of −80 mV. In these experiments, there were an average number of 4 BK channels in each patch.

**Fig. 7.**
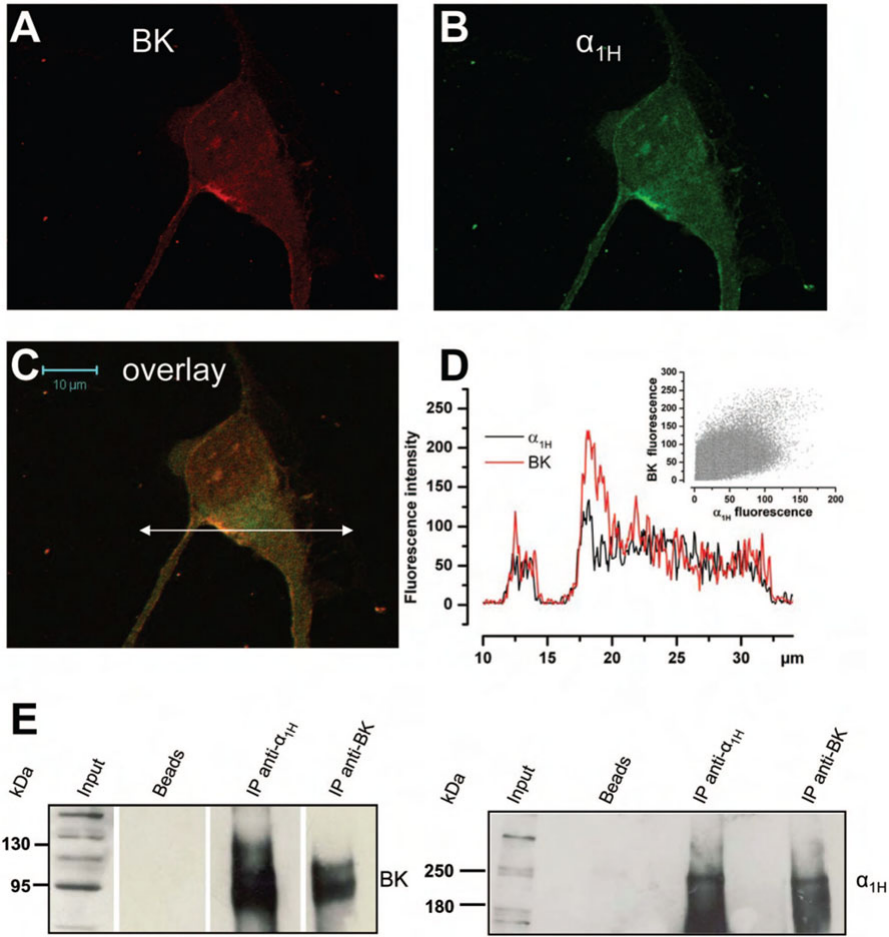
Cav3.2 and BK channels co-localize in the same membrane area and belong to the same molecular complex. (**A–C**) Confocal immunofluorescence images of an LNCaP cell overexpressing Cav3.2 GFP (green) stained with an anti-BK antibody (red). Staining is more pronounced on the plasma membrane for both channels and the overlay shows that there is a co-localization (yellow-orange areas) on plasma membrane areas. Scale bar: 10 µm. (**D**) Representation of both Cav3.2 and BK fluorescence intensities along the horizontal line shown in panel C. Inset: a scattergramme of BK fluorescence *vs* Cav3.2 fluorescence showing a correlation between both channels (Pearson's r = 0.77). (**E**) Western-blot of proteins immunoprecipitated by the anti-Cav3.2 antibody (anti-α_1H_) or the anti-BK antibody. Membranes were revealed with the anti-BK antibody and the anti-α_1H_ (right panel) antibody. Bead lanes contain the beads used during the immunoprecipitation without the protein input.

We therefore investigated whether there could be a functional coupling between Cav3.2 and BK channels in the plasma membrane. In order to study the co-localization of Cav3.2 and BK channels, we carried out single-channel experiments in the cell-attached configuration of the patch-clamp technique. Cells were depolarized with 100 mM KCl in the bath, in order to clamp the membrane potential at a value close to 0 mV. The patch-pipette contained 140 mM NaCl, 5 mM KCl to measure K^+^ currents. As shown in Fig. 6, a depolarization to −20 mV in LNCaP-NE cells induced channel openings. As measured from their unitary conductance, these channels were identified as BK channels (conductance = 150–200 pS). Smaller K^+^ conductances, like the IK channels that we have previously demonstrated to be present and which are activated by cytosolic Ca^2+^ increases in LNCaP cells (Lallet-Daher et al., 2009), were not observed to be activated by Cav3.2 dependent Ca^2+^ entry in our experiments. Channel opening was transiently stimulated immediately after the depolarization. In order to observe T-type Ca^2+^ channel activity, we replaced NaCl by 100 mM CaCl_2_ in the patch-pipette (100 mM CaCl_2_, 5 mM KCl, no NaCl). Under these conditions, a small inward current could be observed just before BK channel openings (Fig. 6C, left panel). We are inclined to think that this small inward current (1 pA) is due to a T-type Ca^2+^ channel opening. Indeed, at such a divalent concentration (100 mM), the single channel conductance of T-type Ca^2+^ channels is close to 7 pS (for a review, see Perez-Reyes and Lory, 2006) and the corresponding single-channel current at −20 mV is expected to be about 1 pA. In addition, as shown in Fig. 6C, this inward current was rapidly inactivated after the onset of depolarization. When T-type Ca^2+^ channels were inactivated by a 10 sec conditioning potential pulse at −40 mV (Fig. 6C, right panel), we did indeed observe that this small inward current was inhibited showing that it is carried by Ca^2+^ entry through Cav3.2. Furthermore, the transient BK channel opening probability (Po) was significantly reduced in LNCaP-NE cells by a previous inactivation of T-type Ca^2+^ channels at −40 mV (Fig. 6C, right panel). Such a functional coupling between Cav3.2 and BK channels in the same patch was observed in all the LNCaP-α_1H_ cell patches and in almost half of the LNCaP-NE ones (46%, *n* = 39 cells). From the average channel density in LNCaP-NE cells displaying a functional coupling (4±0.2 BK channels per patch), we were able to compute the T-type activated BK channel open probability (Fig. 6D). We can observe that the maximum average Po is reached only 30 msec after the onset of depolarization, showing a fast coupling between both Cav3.2 and BK channels. We thus carried out confocal immunofluorescence experiments to study the subcellular localization of these channels. As shown in Fig. 7A–C, confocal experiments using immunofluorescent staining of LNCaP cells overexpressing Cav3.2 channels (LNCaP-NE and LNCaP-α_1H_) show that there is a colocalization of both ion channels, as illustrated by the overlay of fluorescence at both FITC and Rhodamine wavelengths. Fluorescence was particularly strong at the cell periphery (Fig. 7D), indicating that these channels could be expressed together in the same plasma membrane areas. In addition, we performed co-immunoprecipitation studies in order to assess whether Cav3.2 and BK channels belong to the same molecular complex. We show in Fig. 7E that each channel protein could be immunoprecipitated by the other one, indicating that both ion channels could be part of a common protein complex.

We investigated the possibility that Cav3.2 channels could promote Ca^2+^ entry and could thereby activate BK channels. In Fura2 Ca^2+^ imaging experiments, although basal Ca^2+^ was significantly higher in LNCaP-NE cells than in LNCaP-CTL cells (84±5.3 nM (*n* = 50) *vs* 62±1.2 nM (*n* = 61), *P*<0.01), there were no detectable Ca^2+^ oscillations in these cells. In LNCaP-α_1H_ cells, the cytosolic Ca^2+^ concentration was significantly higher (109±3 nM, *n* = 358) than in LNCaP-NE cells (*P*<0.001). Basal Ca^2+^ concentration ([Ca^2+^]i) in both LNCaP-NE and LNCaP-α_1H_ cells was reduced by NiCl_2_ (20 µM, reduction of [Ca^2+^]i: ΔCa = 11±3 nM, *n* = 42) and si-α_1H_1 and si-α_1H_2 (ΔCa = 18±2.5 nM, *n* = 62, *P*<0.001 and 15±1.5 nM, *n* = 43, *P*<0.001, respectively). We carried out current-clamp recordings in zero current conditions to study the functional role of Cav3.2 channels in the Resting Membrane Potential (RMP) setting. BK channels are partially responsible for LNCaP cells RMP (RMP = −32±2 mV, *n* = 15). Indeed, RMP was significantly depolarized by increasing the extracellular K^+^ concentration and by TEA, paxillin (−3.5±1.9 mV, *n* = 27, *P*<0.001) and iberiotoxin (−7±1.8 mV, *n* = 10, *P*<0.001). Similarly, si-hBK strongly depolarized LNCaP cells (−1.7±5.8 mV, *n* = 8, *P*<0.001). In contrast, inhibition of IK or SK channels by siRNAs, apamin or d-tubocurarine did not alter the membrane potential. LNCaP-NE cells were slightly hyperpolarized (−40±0.8 mV, *n* = 10, *P*<0.05) as compared to undifferentiated cells (−32±2 mV), with 0.1 mM EGTA in the patch-pipette. In addition, LNCaP-α_1H_ cells were even more hyperpolarized (−54.2±1 mV, *n* = 32, *P*<0.001). This hyperpolarization was antagonized by both siRNAs raised against Cav3.2 (−41.8±4.4 (*n* = 10) for si-α_1H_1, −29.9±1.9 (*n* = 19) for si-α_1H_2, *P*<0.001) and by 20 µM NiCl_2_ (−37.8±1.3 (*n* = 16), *P*<0.001). When EGTA was increased to 10 mM in the patch-pipette, the RMP of both LNCaP-NE and LNCaP-α_1H_ cells was depolarized to values similar to those measured for LNCaP-CTL cells.

**Fig. 8.**
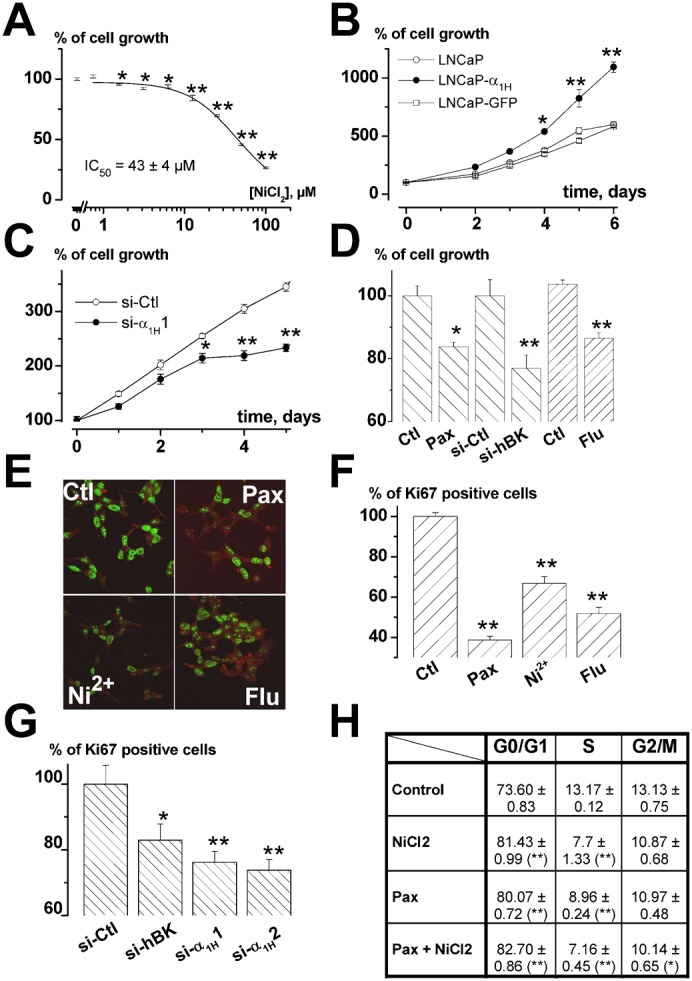
Role of BK and Cav3.2 channels in LNCaP-CTL cell proliferation. (**A**) Inhibition of cell growth (assayed by MTS) induced by a 4-day incubation in various concentrations of NiCl_2_. Data are normalized to the proliferation rates measured in control conditions (100%) as in panel **D**. *n* = 9 per condition. (**B**) Stable overexpression of Cav3.2 stimulated LNCaP cell growth (as assayed by MTS). Results are normalized to DO at t0 (100%) as in panel **C**. *n* = 12 per condition. (C) Inhibition of LNCaP cell growth by si-α_1H_1 (20 nM) measured by MTS. siRNAs were added when seeding the cells. *n* = 12 per condition. (D) Cell growth measured with MTS after 4 days in various channel inhibitors or si-RNAs (Pax: paxillin (10 µM), Flu: flunarizine (10 µM), si-Ctl and si-hBK (20 nM)). *n* = 6 per condition. (**E**) Immunodetection of Ki-67 in LNCaP-CTL cells after 4 days of incubation in various BK and Cav3.2 channel inhibitors (Pax: paxillin 10 µM, Flu: flunarizine 5 µM and Ni^2+^: NiCl_2_ 20 µM) and relative % of cells immunostained with Ki-67 antibody in the presence of these channel inhibitors (**F**) or siRNAs (20 nM) (**G**). (**H**) Table showing the % of cells in each phase of the cell cycle using FACS analysis (G0/G1, S and G2/M). Inhibition of both T-type and BK channels (with 20 µM NiCl_2_ and 10 µM paxillin, respectively) increases the proportion of cells in G0/G1 phase and decreases the proportion of cells in S phase without additive action. This experiment is representative of 3 experiments (for each experiment, *n* = 3, each measured in duplicate). Statistical significance: *P<0.05, **P<0.01, ***P<0.001.

We then investigated whether such a coupling could participate in prostate cancer cell growth. As shown in Fig. 8A using MTS survival assays, cell proliferation was dose-dependently inhibited by NiCl_2_ after 4 days of incubation. At a concentration which blocks Cav3.2 channels at 80% (20 µM), NiCl_2_ induced a 20% cell growth reduction. At concentrations inhibiting Cav3.2 channels, flunarizine (5 µM) or si-α_1H_ (20–50 nM) also induced a similar reduction in cell proliferation (Fig. 8C,D). Furthermore, overexpressing Cav3.2 channels led to a significant stimulation of cell proliferation (Fig. 8B). These results thereby demonstrate that Cav3.2 Ca^2+^ channels are involved in cell proliferation. Similarly, we show that BK channels stimulate LNCaP cell growth. Indeed, paxillin (10 µM) significantly decreased LNCaP cell growth by about 20%. In addition, the inhibitory action of paxillin on proliferation was mimicked by si-hBK, indicating that its action occurs *via* BK channel inhibition (Fig. 8D). Since BK channels are already strongly expressed in LNCaP cells, we did not assess whether the overexpression of BK channels could lead to proliferation stimulation. In order to confirm the results obtained with MTS assay, we also performed Ki-67 immunostaining, which allows the discrimination of quiescent cells in the G0 phase (unstained) from proliferating cells (stained). The number of proliferating cells was determined as the proportion of cells stained by the Ki67 antibody. As illustrated in Fig. 8E–G, the percentage of Ki67 positive cells was reduced by T-type Ca^2+^ channels inhibitors, BK channels inhibitors, si-hBK or si-α_1H_. In addition to increasing the proportion of cells in the G0 phase, a FACS analysis showed that both T-type Ca^2+^ channel inhibition and BK channel inhibition increased the percentage of cells in the G1 phase by 8–10% and decreased the proportion of cells in S and G2/M phases (Fig. 8H). Reduction in cell growth was not due to cell apoptosis since no detectable SubG1 peak was observed with any of the inhibitors or siRNAs used in this study (not shown). Furthermore, there was no additive action of NiCl_2_ (20 µM) and paxillin (10 µM), suggesting that both antagonists decrease cell proliferation *via* common pathways (Fig. 8H). The additive action of siRNAs could not be assessed because of the cytotoxic effects caused by the increased total siRNA concentration.

## Discussion

Our results confirm that BK channels are expressed in LNCaP cells, as previously shown by others (Gessner et al., 2006; Gutierrez et al., 1999) and that most of the voltage-dependent K^+^ current is carried by BK channels in these cells. These BK currents have standard single-channel conductances (about 200 pS in symmetrical K^+^ conditions), but display non-standard Ca^2+^ dependency as previously shown by Gessner et al. (Gessner et al., 2006). Indeed, BK currents can be fully activated in very low concentrations of cytosolic Ca^2+^ (buffered with 10 mM EGTA). In whole-cell configuration with 10 mM EGTA in the recording pipette, BK currents are activated at around −10 mV in LNCaP cells. Such a property has been attributed to a regulating subunit LRCC26 (Yan and Aldrich, 2010). In LNCaP cells, we demonstrate that BK channels maintain the resting membrane potential to values around −30 mV, which are very close to those described elsewhere (Gutierrez et al., 1999; Mariot et al., 2002). In addition, BK channels are sensitive to Ca^2+^ concentration increases. Despite the low density of Cav3.2 channels on the plasma membrane, BK channels were consistently activated by Ca^2+^ entry through Cav3.2 channels, which indicates that there is a specific and functional coupling between both channels in LNCaP cells. However, an activation of IK channels, another Ca^2+^-dependent K^+^ channel expressed in LNCaP cells activated by large increases in cytosolic Ca^2+^ concentration (Lallet-Daher et al., 2009; Parihar et al., 2003), was never observed in response to T-type Ca^2+^ channels activity.

We therefore investigated whether a functional interaction could exist between Cav3.2 and BK channels. There is evidence showing co-localization and coupling between different voltage-dependent Ca^2+^ channels and Ca^2+^-dependent K^+^ channels. For instance, L-type Ca^2+^ channels have been shown, using single-channel experiments, to be specifically coupled to SK channels (Marrion and Tavalin, 1998). In addition, T-type Ca^2+^ channels have been shown to be coupled to small conductance SK channels in dopaminergic neurons (Wolfart and Roeper, 2002). Such functional couplings between BK and other voltage-dependent Ca^2+^ channels have been demonstrated in various cell types, such as L- and Q-type channels in adrenal chromaffin cells (Prakriya and Lingle, 1999), L-type and N-type channels in neocortical pyramidal neurons (Sun et al., 2003) or the active zones of hair cells (Issa and Hudspeth, 1994; Samaranayake et al., 2004). Co-localization and physical interaction have been demonstrated between BK channels and L-type Cav1.2 channels in rat brain and adrenal chromaffin cells (Wolfart and Roeper, 2002; Grunnet and Kaufmann, 2004; Berkefeld et al., 2006). In our experiments, because functional coupling was frequently observed in cell-attached patch single channel experiments (in half of the cell-attached patches in neuroendocrine LNCaP cells), we suggest that there is a co-localization of both channels. Firstly, the location of BK channels in clusters (50% of BK channels are pooled in only 17% of the plasma membrane surface) is a strong argument for a functional coupling with other ion channels. Furthermore, coupling was observed essentially in patches displaying 3 or more single BK channel opening levels, which means that Cav3.2 channels are probably located in BK channel clusters. In addition, our co-immunoprecipitation and immunofluorescence experiments suggest that both channels belong to the same macromolecular complexes.

The activation of BK channels by Cav3.2 channels was observed not only in a Cav3.2 overexpressing cell model (LNCaP-α_1H_), but also in cells endogenously expressing moderate levels of Cav3.2 channels (from 0.3 pA/pF (1 channel/30 µm^2^) to 1 pA/pF (1 channel/10 µm^2^)). Despite such a low Cav3.2 channel density, BK channels were activated in all LNCaP cells expressing functional Cav3.2 currents. Since endogenous expression of Cav3.2 channels is not associated with calcium oscillations in LNCaP cells, it is likely that Cav3.2 channel activity only allows calcium increases restricted to small areas underneath the plasma membrane. These local Ca^2+^ increases may in turn activate nearby BK channels. In prostate cancer cells, such Ca^2+^ entry would occur at a resting membrane potential (RMP) window, as shown previously for Cav3.2 channels in LNCaP cells (Mariot et al., 2002), in other endogenously expressing cell models (Bijlenga et al., 2000) and in overexpressing cell models (Xie et al., 2007). In prostate cancer cells, this RMP window is probably set by BK and Cav3.2 channel coupling. Indeed, inhibiting BK channels depolarized the RMP to close to 0 mV, showing that they are the main ion channels involved in this function. In addition, overexpressing Cav3.2 channels surprisingly led to a more hyperpolarized RMP, whereas blocking the Cav3.2 expression or function produced a depolarized RMP. Therefore, Cav3.2 channels are able, through BK channel activation, to shift the RMP towards negative values. It is therefore probable that RMP automatically equilibrates at around the optimum value for generating a window Ca^2+^ current through Cav3.2 channels, thereby producing a steady Ca^2+^ entry. If RMP drifts towards more negative values (hyperpolarization), this will close Cav3.2 channels. This will in turn decrease basal Ca^2+^ entry and thereby reduce the cytosolic Ca^2+^ concentration underneath the plasma membrane. This would lead to reduced BK channel activation and thus to a membrane depolarization that may reopen Cav3.2 channels once the optimal RMP has been reached.

In neuronal cells, functional coupling between voltage-dependent Ca^2+^ channels and Ca^2+^-dependent K^+^ channels has been shown to participate in action potential repolarization (Sun et al., 2003) or burst firing (Wolfart and Roeper, 2002; Swensen and Bean, 2003). We investigated the potential role of this coupling in non-excitable prostate cancer cells proliferation. The role of BK channels in proliferation has previously been studied in different cell models and noticeably in prostate cancer cells, where they would appear to be either stimulatory or inhibitory or negligible according to the cell type or the cell line. For example, in osteosarcoma, BK-silencing using an siRNA strategy induced *in vivo* tumorigenesis (Cambien et al., 2008). In breast cancer cells, whilst their expression is correlated to the different phases of the cell cycle, their inhibition by iberiotoxin was not correlated to any changes in proliferation (Ouadid-Ahidouch et al., 2004; Roger et al., 2004). In addition, to add even more complexity, their stimulation by tamoxifen could promote cell proliferation (Coiret et al., 2007), an action inhibited by BK channel blockade. In gliomas, BK channels have been shown to be upregulated in high grades of the diseases (Liu et al., 2002). Furthermore, their inhibition leads to reduced glioma cell proliferation (Weaver et al., 2004). In the prostate, it has recently been shown that BK channels are overexpressed in cancer and that their inhibition reduces cell proliferation (Bloch et al., 2007; Oeggerli et al., 2012). Similarly, a role for T-type Ca^2+^ channels in proliferation and cancer progression has been suggested in various cell types (for reviews, see Lory et al., 2006; Panner and Wurster, 2006), such as breast cancer cell lines (Taylor et al., 2008), oesophageal cancer (Lu et al., 2008) or gliomas (Panner et al., 2005). Our results obtained using FACS analysis of the cell cycle, cell survival and Ki67 expression, point to a joint role of both BK and Cav3.2 channels in cell proliferation. Indeed, their pharmacological inhibition or their downregulation by specific siRNAs decreased the proportion of cells in the S-phase of the cell cycle, slowing cell growth and reducing the proportion of cells expressing Ki-67, which is a known marker of proliferating cells. Besides, no additive inhibition was observed when T-type Ca^2+^ channels and BK channels were simultaneously pharmacologically inhibited, showing that they are involved in a common pathway implicated in cell proliferation. In addition, we have observed that BK channels, as previously demonstrated for Cav3.2 channels (Gackière et al., 2006), are involved in Prostatic Acid Phosphatase secretion (not shown). This allows us to speculate that the role of BK and Cav3.2 channels in proliferation may be either direct, by activating transcription factors relying on Ca^2+^ signalling as previously demonstrated for TRPC6 channels (Thebault et al., 2006), or indirect, by promoting the secretion of mitogenic factors which in turn could activate cell proliferation.

In summary, we have shown here that both Cav3.2 and BK channels localize in same plasma membrane areas and may be part of a common molecular complex. Through such an interaction, they might regulate prostate cell proliferation. Such a role played by coupled BK and Cav3.2 channels could explain previously published discrepancies relative to the role of either BK or Cav3.2 channels in proliferation (for reviews, see Lory et al., 2006; Panner and Wurster, 2006). It is therefore likely that the role of each channel in cell proliferation depends on a concerted action with other channels or partner proteins.

## Materials and Methods

### Cell culture and treatments

LNCaP cells were purchased from the American Type Culture Collection and grown as recommended in RPMI 1640 (Gibco, Life Technology, France) supplemented with 10% fetal bovine serum (FBS, Seromed, Poly-Labo, Strasbourg, France), and 2 mM L-glutamine (Sigma, L'Isle d'Abeau, France). Cells were routinely grown in 75 cm^2^ flasks (Nunc, Poly-Labo, France) in a humidified atmosphere at 37°C (95% air–5% CO_2_). For electrophysiological and imaging studies, cells were detached from their support using trypsin–EDTA for two minutes, centrifuged and sub-cultured in Petri dishes (Nunc) and on glass coverslips for imaging studies only. The culture medium was then changed every three days. In order to induce neuroendocrine differentiation, LNCaP cells were cultured with 1 mM dibutyryl cyclic-AMP (Bt_2_cAMP) and 100 µM isobutylmethylxanthine (IBMX) for 3–6 days. Stable cell lines expressing Cav3.2 (α_1H_) channel (LNCaP-α_1H_), or α_1H_-GFP fusion protein (LNCaP-α_1H_GFP) were designed as previously reported (Gackière et al., 2008).

### Fluorescence imaging

Fluorescence imaging was carried out in HBSS (Hank's Balanced Salt Solution) containing 142 mM NaCl, 5.6 mM KCl, 1 mM MgCl_2_, 2 mM CaCl_2_, 0.34 mM Na_2_HPO_4_, 0.44 mM KH_2_PO_4_, 10 mM HEPES and 5.6 mM glucose. The osmolarity and pH of external solutions were adjusted to 310 mOsm.l^−1^ and 7.4, respectively. Cytosolic Ca^2+^ concentration was measured using Fura2-loaded cells (2 µM) as described elsewhere (Gackière et al., 2006). The intracellular Ca^2+^ concentration was derived from the ratio of the fluorescence intensities for each of the excitation wavelengths (F340/F380) and from the Grynkiewicz et al. equation (Grynkiewicz et al., 1985). The cells were continuously perfused with the HBSS solution and chemicals were added *via* a perfusion system.

### Electrophysiological recordings

Whole-cell patch-clamp recordings (Hamill et al., 1981) were performed using a RK-300 patch-clamp amplifier (Biologic, Grenoble, France) as previously described (Gackière et al., 2008). Whole-cell membrane currents were measured in the voltage-clamp mode after the previous cancelation of series resistance (Rs value was usually 5 MOhm when breaking into whole-cell configuration and kept stable for the first 10 minutes of each experiment). Unless otherwise specified, the bath medium used for whole-cell and outside-out experiments was HBSS. For cell-attached single-channel recordings, the bath medium contained 100 mM KCl, 45 mM NaCl, 1 mM MgCl_2_, 10 mM HEPES, 5.6 mM glucose and 2 mM CaCl_2_. We previously conducted current-clamp experiments to determine that this solution induced a membrane depolarization to 0 mV. The osmolarity and pH of the external buffers were adjusted to 310 mOsm.l^−1^ and 7.4, respectively. For whole-cell or outside-out single-channel experiments, the recording pipettes were filled with a solution containing 130 mM K-Gluconate, 10 mM NaCl, 10 mM HEPES, 1 mM MgCl_2_, with 0.1 to 10 mM EGTA. For cell-attached single-channel experiments, the pipette solution contained standard HBSS or a medium containing 100 mM CaCl_2_, 10 mM HEPES and 5 mM KCl. Osmolarity and pH were adjusted to 290 mOsm.l^−1^ and 7.2, respectively.

Junction potential was cancelled when the patch-pipette was dipped in the bath solution (standard HBSS containing 5 mM K^+^ and 145 mM Na^+^) close to the cell before sealing the cell membrane. Using JPCalc software we calculated that the greatest variation in liquid junction potential that could occur in our experiments (when bath medium was changed from 145 mM Na^+^ and 5 mM K^+^ to 0 mM Na^+^ and 150 mM K^+^) was −3.7 mV. The chloride concentration was kept constant during perfusion. Resting membrane potential (RMP) values (measured in zero current conditions) compensated for these calculated values (RMP  =  Recorded membrane potential – Liquid Junction Potential) in our experiments.

### siRNAs design and cell preparation

Small interfering RNAs against the human coding sequence of Cav3.2 (α_1H_) channels (Genebank accession # NM-021098.2) were designed and two selective sequences, referred to as si-α_1H_1 and si-α_1H_2, were selected to knock down the expression of T-type α_1H_ Ca^2+^ channels. We have previously validated these siRNAs in LNCaP cells (Gackière et al., 2008). In addition, siRNAs against human forms of BK channels (referred to as si-hBK, Genebank accession # HSU11717) and IK1 channels (referred to as si-hIK1, Genebank accession # NM_002250) were used to downregulate the expression of both large and intermediate conductance Ca^2+^-dependent K^+^ currents, respectively. We have also previously validated the siRNAs directed against the human form of KCa3.1 channels in LNCaP cells (Lallet-Daher et al., 2009). The siRNAs used in this study included a non-specific control siRNA (si-Ctl) with at least 4 mismatches to any human genes. Sense sequences of siRNAs were 5′-UAGCGACUAAACACAUCAA(dTdT)-3′ (si-Ctl), 5′-ACGUGAGCAUGCUGGUAAU(dTdT)-3′ (si-α_1H_1, position 311–329 from ATG), 5′-AGAUGGCCGUGGCGUCUAU(dTdT)-3′ (si-α_1H_2, position 2166–2184 from ATG), 5′-GAGUCCUGGUUGUCUUAGU(dTdT)-3′ (si-hBK, position 485–503 from ATG), and 5′-GUUCGUGGCCAAGCUUUACA(dTdT)-3′ (si-hIK1, position 975–994 from ATG). siRNAs were purchased from Dharmacon (France).

LNCaP-CTL and LNCaP-α_1H_ cells were transfected with either 5 or 20 nM siRNA si-α_1H_1, si-α_1H_2, si-hBK, si-hIK1 or si-Ctl using HiPerFect Transfection Reagent (Qiagen) as described previously (Gackière et al., 2008).

### Analysis of the BK and IK channel gene expression (RT-PCR)

RT-PCR was carried out as previously described (Gackière et al., 2008). The PCR primers used to amplify the 794 bp KCa1.1 amplicon (accession number: NM_001014797.1) were 5′- CAGACACTGACTGGCAGAGT-3′ (forward) and 5′-TGACGTCATCCCGGTCCTTGTGCA-3′ (reverse), and those used to amplify the 234 bp GAPDH amplicon (accession number: NM_002046.3) were 5′-TTCACCACCATGGAGAAGGC-3′ (forward) and 5′-GGCATGGACTGTGGTCATGA-3′ (reverse). The PCR primers used to amplify the 578 bp KCa3.1 amplicon (accession number: NM_002250) were 5′-TCCAGCAGCCATCAGCAAGCCA-3′ (forward) and 5′-GCTGGAGTTTAACAAGGCAGA-3′ (reverse).

### Western blotting and co-immunoprecipitation

After being washed in phosphate buffered saline (PBS), cells were collected by scraping in a lysis buffer (Triton X-100 1%, Na deoxycholate 1%, NaCl 150 mM, PO_4_NaK 10 mM, pH 7.2) with an anti-protease cocktail and incubated on ice for 45 min. The lysates were centrifuged at 12000 G for 10 min at 4°C. The protein concentration of the supernatant was determined by BCA assay (Pierce Chemical Company). For co-immunoprecipitation, a mixture containing protein A/G PLUS agarose beads (Santa Cruz, Biotechnology) and 1 µg anti-BK (or anti-α_1H_) antibody was incubated under rotation for 1 h at 4°C. An equal amount of cell extract (500 µg) and protease inhibitor were added to the total volume and incubated under rotation overnight at 4°C. After washing, the beads–antibody–proteins complex was re-suspended in SDS sample buffer for 30 min at 37°C. The immune complexes were separated by electrophoresis on 6 or 8% SDS-PAGE gels. Western-blot analysis of protein expression was designed as described elsewhere (Gackière et al., 2008) using the same primary antibodies as in immunoprecipitation: anti-α_1H_ polyclonal antibody (1/200, rabbit, Santa Cruz) and anti-BK polyclonal antibody (1/500, rabbit, Alomone).

Cell extract (50 µg) without immunoprecipitation was also used as a source of marker proteins (Input) and was submitted directly to SDS-PAGE and immunoblotting as presented in the figures.

### Viability test

Cell viability was assessed by a colorimetric method (CellTiter 96 Aqueous Non-Radioactive Cell Proliferation Assay, Promega, USA) according to the manufacturer's instructions. Cells were grown on 24 well plates. Each mean represents the averaged value of 6–12 different measurements. Each experiment is representative of at least three independent experiments.

### Cell cycle analysis

For each condition, cells were grown in three 60-mm dishes and each measurement was done in duplicate. Drugs or siRNAs were applied as described above. After treatments, cells were trypsinized, harvested and resuspended in 0.2 ml sterile PBS. 1 ml of cold 70% ethanol was added to cell suspensions while vortexing. Samples were centrifuged, washed in sterile PBS and then incubated with ribonuclease (2 µg/ml) for 15 min at room temperature. Propidium iodide (25 µg/ml final in PBS–Triton X-100 0.1%) was then added and allowed to incubate for an additional 30 min at room temperature. DNA content was measured by exciting propidium iodide at 488 nm and measuring the emission at 520 nm, using a flow cytometer (Beckman coulter Epics XL4-MCL with Expo32 acquisition). Data analysis was carried out using Multicycle for Windows (Phoenix Flow system). Each experiment is representative of at least three independent experiments.

### Immunostaining and confocal analysis

Immunostaining and confocal observations were carried out as previously described (Gackière et al., 2008), using a Zeiss LSM 510 confocal microscope (Carl Zeiss, Le Pecq, France) connected to a Zeiss Axiovert 200 M with a ×63 oil-immersion objective lens (numerical aperture 1.4). Anti-Ki-67 antibody was used to evaluate the percentage of proliferating cells (1/100, rabbit, Abcam). At least 500 cells per slide and three slides per condition were double-blind counted. Co-localization of BK channels and Cav3.2 channels was studied using both anti-α_1H_ polyclonal antibody (1/200, rabbit, Santa Cruz) and anti-BK polyclonal antibody (1/500, rabbit, Alomone).

### Chemicals

All chemicals were purchased from Sigma, except for Fura2-AM, which was bought from Calbiochem (France Biochem, Meudon, France).

### Statistical analysis

Each average current/voltage relationship shown in this article corresponds to the mean of at least 10 cells and is representative of at least 3 independent experiments. Plots were produced using Origin 7.0 (Microcal Software, Inc., Northampton, MA). Results are expressed as mean ± s.e.m. Statistical analyses were performed using unpaired t-tests (for comparing two groups) or ANOVA tests, followed by either Dunnett (for multiple control *vs* test comparisons) or Student-Newman-Keuls (for multiple comparisons) post-tests. Contingency tables were analyzed using Fisher's test. Differences were considered significant with *P<0.05, **P<0.01, ***P<0.001.
